# Prolonged mitotic arrest induces a caspase-dependent DNA damage response at telomeres that determines cell survival

**DOI:** 10.1038/srep26766

**Published:** 2016-05-27

**Authors:** Karolina O. Hain, Didier J. Colin, Shubhra Rastogi, Lindsey A. Allan, Paul R. Clarke

**Affiliations:** 1 Division of Cancer Research, School of Medicine, University of Dundee, Jacqui Wood Cancer Centre, Ninewells Hospital, Dundee DD1 9SY, Scotland, UK

## Abstract

A delay in the completion of metaphase induces a stress response that inhibits further cell proliferation or induces apoptosis. This response is thought to protect against genomic instability and is important for the effects of anti-mitotic cancer drugs. Here, we show that mitotic arrest induces a caspase-dependent DNA damage response (DDR) at telomeres in non-apoptotic cells. This pathway is under the control of Mcl-1 and other Bcl-2 family proteins and requires caspase-9, caspase-3/7 and the endonuclease CAD/DFF40. The gradual caspase-dependent loss of the shelterin complex protein TRF2 from telomeres promotes a DDR that involves DNA-dependent protein kinase (DNA-PK). Suppression of mitotic telomere damage by enhanced expression of TRF2, or the inhibition of either caspase-3/7 or DNA-PK during mitotic arrest, promotes subsequent cell survival. Thus, we demonstrate that mitotic stress is characterised by the sub-apoptotic activation of a classical caspase pathway, which promotes telomere deprotection, activates DNA damage signalling, and determines cell fate in response to a prolonged delay in mitosis.

The fidelity of mitosis is maintained by the mitotic or spindle assembly checkpoint, which restrains anaphase until all chromosomes are properly attached to spindle microtubules^1^. Cells held in mitosis for a prolonged period, for instance by chemotherapeutic microtubule poisons, can undergo a variety of fates, including death by apoptosis^2^,^3^,^4^. During mitotic arrest apoptosis is promoted by degradation of the anti-apoptotic protein Mcl-1 after its phosphorylation by cyclin B1-CDK1^5^,^6^. Slow degradation of cyclin B1 even though the checkpoint is active can lead eventually to cells slipping from mitotic arrest^7^, but such cells can subsequently undergo cell cycle arrest in G1 or apoptosis^8^,^9^. These responses may select against cells that have failed to undergo chromosome segregation on schedule and which are therefore likely to produce daughter cells that carry aberrant chromosomes.

The nature of the stress signal generated by mitotic arrest and its relationship to the mechanism of apoptosis have been unknown. There is an accumulation of DNA damage in cells arrested in mitosis, as evidenced by increase in phosphorylated histone H2AX (γH2AX)^10^,^11^. Other work has indicated that deprotection of telomeres during mitotic arrest initiates a DNA damage response (DDR) that controls subsequent cell cycle progression and cell death^10^. Evidence has also been provided that a widespread DDR induced by mitotic arrest is a consequence of caspase activation, suggesting that mitotic arrest induces partial apoptosis^12^, although it has been unclear if this process is related to telomere deprotection^13^.

In this report, we demonstrate that a mitotic DDR at telomeres depends on sub-apoptotic activation of the classical caspase-9/3/7 pathway under the control of Mcl-1 and other Bcl-2 family proteins. This mitotic DDR requires DNA-PK and involves caspase-dependent loss of TRF2 from telomeres. We show that suppression of this response during mitotic arrest promotes subsequent cell survival. This mitotic stress pathway is likely to be important for the response of cancer cells to chemotherapeutic drugs that disrupt mitosis.

## Results and Discussion

### Mitotic DNA damage at telomeres in non-apoptotic cells requires caspase activity and is under the control of Bcl-2 family proteins

**Figure 1:**
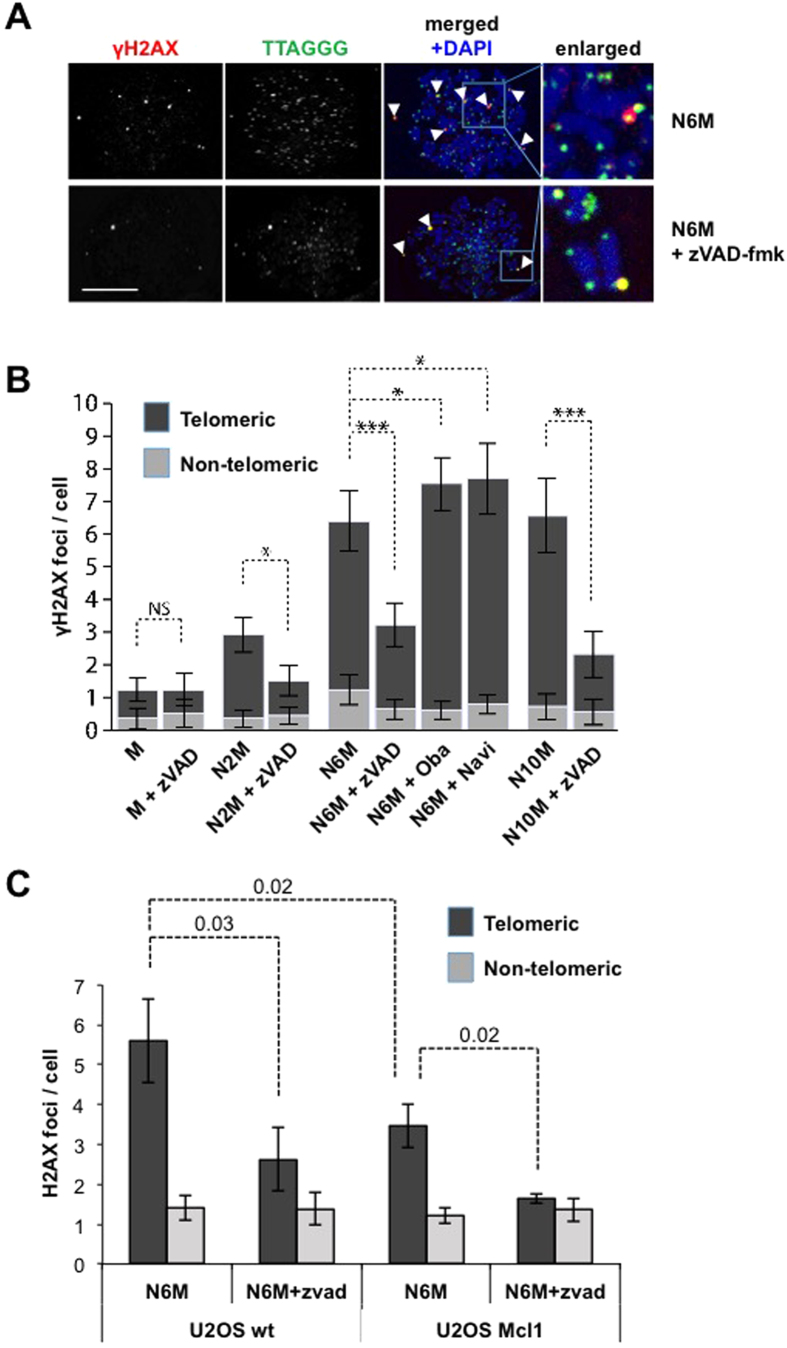
Mitotic telomere damage is caspase dependent. (**A**) Mitotic U2OS cells synchronised for 2 h in 100 ng/ml nocodazole were collected and replated in nocodazole for further 4 h (N6M). Cells were also co-treated with z-VAD-fmk (20 μM). Mitotic cells were immunostained using an anti-γH2AX antibody and telomeres were visualised using a telomere probe (TTAGGG). DNA was stained with DAPI. Scale bar, 10 μm. (**B)** Mitotic U2OS cells were synchronised for a total of 2, 6 or 10 h (N2M, N6M and N10M). Untreated mitotic cells (M) were collected from asynchronous cultures. Cells were also co-treated with z-VAD-fmk (20 μM), Obatoclax (500 nM) or Navitoclax (50 nM). The mean number of γH2AX foci counted in at least 200 cells per treatment are shown +/− SD. ns, non-significant, *p < 0.05, **p < 0.01 and ***p < 0.001. (**C)** Mitotic U2OS cells (U2OS wt) and cells stably expressing Mcl-1 (U2OS Mcl1) were synchronised in mitosis for a total of 6h (N6M) using nocodazole. The mean number of γH2AX foci counted in at least 200 cells per treatment are shown+/− SD.

Foci of histone H2AX phosphorylated on Ser139 (γH2AX), which marks sites of DNA damage on chromosomes, were induced in human osteosarcoma U2OS cells synchronised in the period of mitotic arrest by collection of rounded-up mitotic cells after treatment of an asynchronous culture for 2 h with nocodazole and replating for a further 2–8 h in nocodazole^5^. Note that these cells were not pre-synchronised with any agent such as thymidine that causes DNA damage. The γH2AX foci in mitotically-arrested cells were predominantly localised at telomeres detected by an oligonucleotide probe (Fig. 1A), consistent with previous findings by Hayashi *et al.*^10^. Cells arrested in mitosis for 2 or 6 h showed a time-dependent increase in the mean number of γH2AX foci formed at telomeres compared to normal mitotic cells (Fig. 1B). We found that the formation of these foci was significantly inhibited by the caspase inhibitor zVAD-fmk (Fig. 1A,B) and was enhanced by treatment of the arrested cells with either the Mcl-1/Bcl-2/Bcl-x_L_ inhibitor Obatoclax (GX15-070)^14^ or another BH3 mimetic Navitoclax (ABT-263) that inhibits Bcl-2 and Bcl-x_L_ but not Mcl-1^15^ (Fig. 1B). Conversely, telomeric γH2AX foci were specifically reduced when Mcl-1 was overexpressed (Fig. 1C).

Caspase-dependent telomeric γH2AX foci were also formed during mitotic arrest in a variety of other mammalian cell lines including human A549 cells that use telomerase for telomere maintenance rather than the ALT pathway used in U2OS cells (Supplementary Fig. 1A). Cells arrested in mitosis by expression of the checkpoint protein MAD2 also exhibited a caspase-dependent DDR, showing that it was not due to the microtubule poison per se (Supplementary Fig. 1B). Thus, the time-dependent formation of γH2AX foci at telomeres during mitotic arrest requires caspase activity and is under the control of Bcl-2 family proteins.

### Mitotic arrest induces a time-dependent increase in sub-apoptotic caspase-3/7 activity

**Figure 2:**
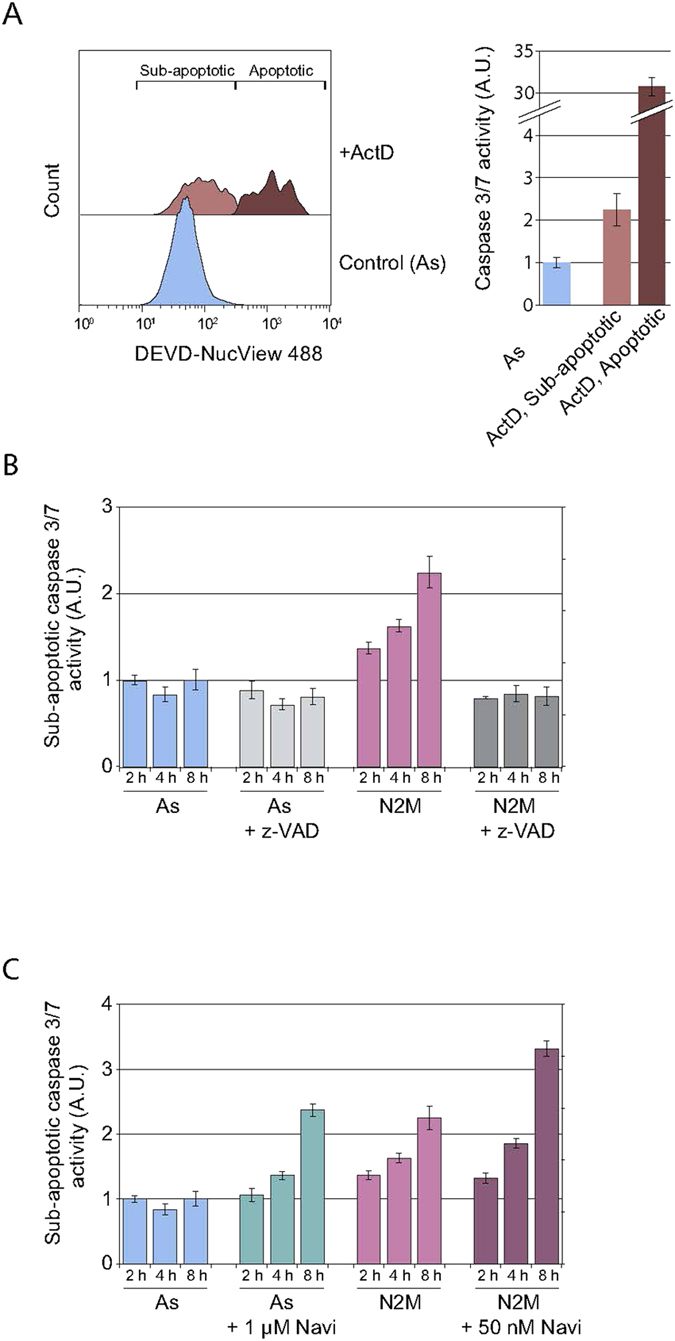
Sub-apoptotic caspase activation during mitotic arrest. (**A)** Induction of apoptotic and sub-apoptotic caspase-3/7 activity. Asynchronous U2OS cells untreated (As) or pre-treated with actinomycin D for 24 h (ActD) were incubated with the fluorescent caspase-3/7 substrate DEVD-NucView 488 (NucView) and analysed by flow cytometry. Relative mean fluorescence intensities in sub-apoptotic and apoptotic cells ± SD from 3 independent experiments (right), one of which is shown (left). (**B**,**C)** Induction of sub-apoptotic caspase-3/7 activity by mitotic arrest. Asynchronous cells (As) or cells arrested in mitosis for 2 h with nocodazole (N2M) were incubated with (**B**) zVAD-fmk (zVAD) or (**C**) Navitoclax (Navi) or for a further 4 h with further addition of nocodazole in the case of N2M samples. Caspase-3/7 activity was assayed by flow cytometry using DEVD-NucView 488. Relative mean fluorescence intensities in non-apoptotic cells ± SD from 3 independent experiments are shown.

Since γH2AX foci were induced in a caspase-dependent manner in mitotic-arrested cells, we inferred that caspase-3/7 activity must be increased during mitotic arrest even in cells that do not subsequently undergo apoptosis. To examine caspase-3/7 activity directly in such cells, we used a highly sensitive assay (NucView) in which a cumulative, stable chromatin-binding fluorescent product is generated by caspase-3/7 cleavage and is analysed by flow cytometry (Supplementary Fig. 2A). In preliminary experiments, we found that induction of caspase-3/7 activity associated with apoptosis in an asynchronous culture of U2OS cells by actinomycin D not only generated cells with relatively high levels of caspase activity associated with apoptosis, but also increased sub-apoptotic levels of caspase activity in other cells (Fig. 2A, Supplementary Fig. 2B). In cells arrested in mitosis for a total of 2–10 h by nocodazole treatment there was a shift in the basal peak of fluorescence to a higher fluorescence that was dependent on the time of the mitotic arrest (Fig. 2B, Supplementary Fig. 2D). The mean fluorescence of this peak reached about 2-fold above basal fluorescence after a total of 10 h arrest although it was still about 10-fold less than in apoptotic cells (Supplementary Fig. S2D). This increase in fluorescence was completely prevented by the caspase inhibitor zVAD-fmk (Fig. 2B, Supplementary Fig. 2C,D). These results indicate that essentially all mitotically-arrested cells had low but measurable levels of sub-apoptotic caspase activity that increased with the period of mitotic arrest.

Treatment of mitotic-arrested cells with 50 nM Navitoclax, which selectively inhibits Bcl-2 and Bcl-x_L_, produced a population of apoptotic cells with greatly increased fluorescence, as expected, and also a population with increased sub-apoptotic caspase-3/7 activity (Fig. 2C, Supplementary Fig. 2C). By contrast, up to 200 nM Navitoclax produced very little effect on asynchronous (predominantly interphase) cells (Supplementary Fig. S2C), confirming the enhanced sensitivity of mitotic-arrested cells to this reagent^16^. Sub-apoptotic caspase activity was enhanced 2–3 fold by treatment of the mitotic-arrested cells with 50 nM Navitoclax and was completely inhibited by zVAD-fmk (Supplementary Fig. S2D). Sub-apoptotic caspase-3/7 activation and subsequent telomeric damage are, therefore, under the sensitive control of Bcl-2/Bcl-x_L_ during mitotic arrest when Mcl-1 is progressively destroyed^5^,^6^. These results demonstrate that cells undergoing transient mitotic stress exhibit low-level caspase activation leading to telomere damage, whereas mitotic arrest results in apoptosis only when a higher threshold of caspase activity is achieved.

### Mitotic telomere damage requires the caspase-9/7 pathway and the endonuclease CAD/DFF40

**Figure 3:**
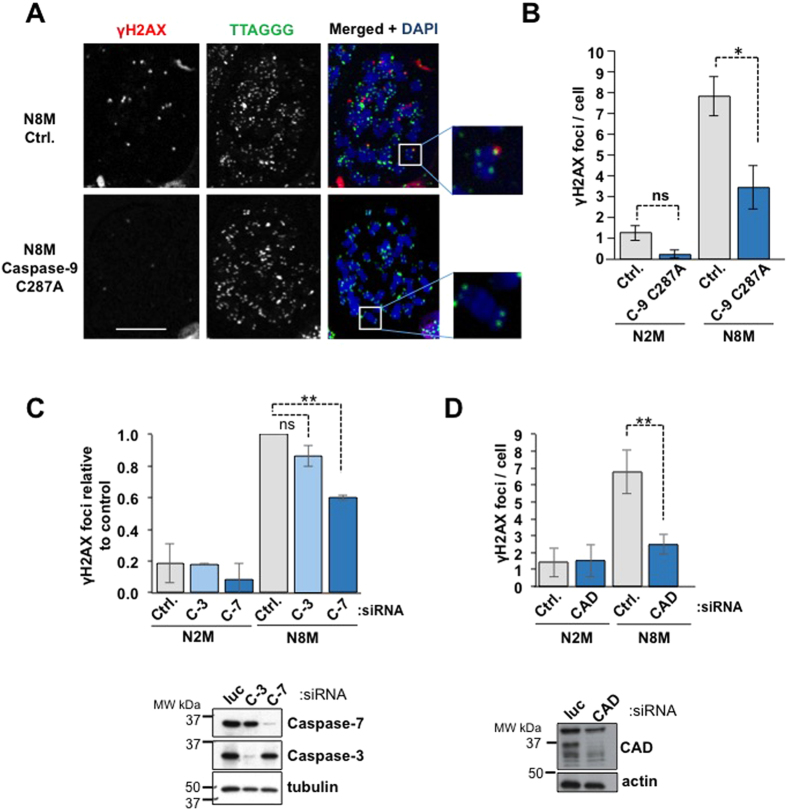
Mitotic telomere damage is dependent on caspase-9, caspase-7 and the endonuclease CAD. (**A**,**B**) U2OS cells including those expressing a dominant inhibitory caspase-9 mutant (C287A) were treated with nocodazole and at least 50 cells were analysed in each of three independent experiments. Representative microscopic fields for (**B)** are shown in (**A**). (**C**,**D)** U2OS cells were transfected with siRNA oligos targeting caspase-3 (C-3), caspase-7 (C-7) (**C)**, the endonuclease CAD (**D**) or a control siRNA (luciferase, Ctrl). The mean number of γH2AX foci per cell is represented in the graph (data from three independent experiments in which at least 50 cells were analysed). Lower panels, Western blots with the indicated antibodies showing depletion of the targeted proteins.

We found that expression of catalytically inactive caspase-9 C287A, which acts as a dominant inhibitor of caspase-3/7 activation during mitosis^17^, significantly reduced the number of γH2AX foci in mitotic-arrested cells (Fig. 3A,B). In addition, the number of foci was significantly reduced by depletion of caspase-7 (Fig. 3C). Caspases-3 and -7 induce widespread internucleosomal DNA strand breaks during apoptosis by activation of the endonuclease CAD/DFF40 through cleavage of its protein inhibitor ICAD/DFF44^18^. We found that siRNA-mediated depletion of CAD strongly inhibited the formation of γH2AX foci in mitotic-arrested cells by almost 70% (Fig. 3D). These results demonstrate that the mitotic telomere damage response is dependent on CAD activated mainly by sub-apoptotic caspase-7 activity downstream of caspase-9. They also strongly suggest that the response is initiated at DNA strand breaks catalysed by the endonuclease activity of CAD.

### The mitotic damage response involves caspase and CAD-dependent TRF2 loss from telomeres

**Figure 4:**
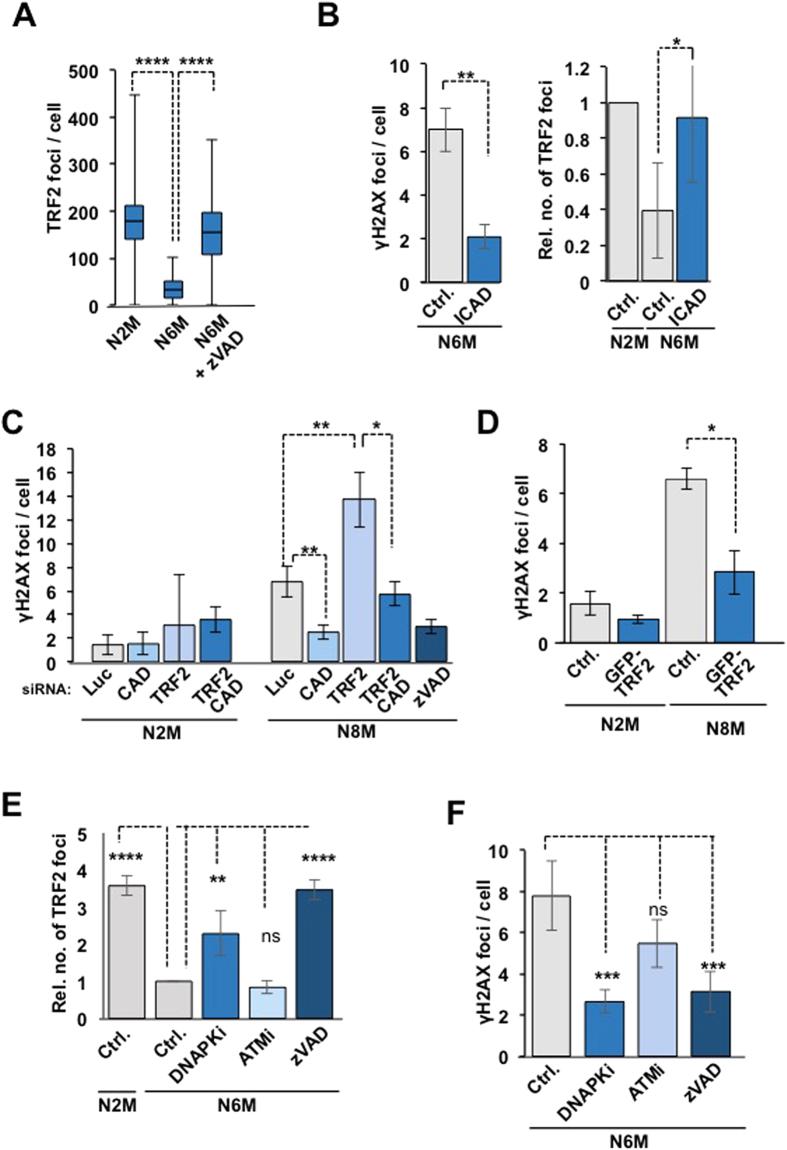
TRF2 loss from telomeres during mitotic arrest is caspase-dependent and promotes damage signalling. (**A)** Caspase-dependent TRF2 loss is determined by the period of mitotic arrest. U2OS cells were treated with nocodazole for 2 h +/− zVAD-fmk. Mitotic cells were collected (N2M) or replated for a further 4 h (N6M). Levels of significance were determined with a two sample z-test. ****p < 0.0001 (**B)** Expression of YFP-ICAD (D117E/D224E) prevents the formation of γH2AX foci (left) and the loss of TRF2 (right) during mitotic arrest. The graphs show means +/− SD from three independent experiments. *p < 0.05, **p < 0.01. (**C)** Depletion of TRF2 enhances the CAD-dependent formation of γH2AX foci. U2OS cells were transfected with siRNA against CAD or TRF2 or CAD and TRF2 simultaneously. The graph shows mean numbers of foci per cell +/− SD from three independent experiments. *p < 0.05, **p < 0.01. (**D)** Overexpression of TRF2 prevents the formation of γH2AX foci during mitotic arrest. Mitotic U2OS cells expressing GFP-TRF2 or GFP alone treated with nocodazole were collected at indicated timepoints. The graph shows mean numbers of foci per cell +/− SD from three independent experiments; *p < 0.05. (**E**,**F)** The loss of TRF2 (**E**) and formation of γH2AX foci (**F**) during mitotic arrest is DNA-PK dependent. Asynchronous cultures were treated with nocodazole and chemical inhibitors of DNA-PK (NU7441, PKi), ATM (KU55933, ATMi) or Caspase-3/7 (zVAD-fmk, zVAD). After 2 h mitotic cells (N2M) were replated in nocodazole +inhibitor for 4 h (N6M). The graphs show means +/− SD of three independent experiments. Differences from N6M samples without enzyme inhibitors are indicated; *p < 0.05, **p < 0.01, ***p < 0.001, ****p < 0.0001; ns, not significant.

The formation of telomeric γH2AX foci during a prolonged mitotic arrest is coincident with loss of the shelterin complex protein TRF2 from telomeres^10^. We confirmed that there was a time-dependent loss of TRF2 at telomeres during mitotic arrest (Fig. 4A, Supplementary Fig. 3A) while telomeres were maintained (Supplementary Fig. 3B). Importantly, the loss of TRF2 from telomeres was strongly inhibited by zVAD-fmk (Fig. 4A). Expression of a non-cleavable ICAD mutant (D117E, D224E) that inhibits CAD independently of caspase activity^18^ also both reduced the formation of γH2AX foci and prevented the loss of TRF2 from telomeres (Fig. 4B). Interestingly, only 13% of the γH2AX foci were also positive for TRF2 in cells arrested in mitosis for 6 h (Supplementary Fig. S3A), consistent with the promotion of γH2AX formation by telomere deprotection. Indeed, depletion of TRF2 with siRNA^19^ before mitosis (Fig. S3C) caused a strong increase in γH2AX foci formed during mitotic arrest that was still prevented by co-depletion of CAD or addition of zVAD-fmk (Fig. 4C). Thus, telomere deprotection due to TRF2 loss alone is insufficient to generate the mitotic DDR at telomeres and this response also requires CAD-dependent DNA damage. Conversely, when we maintained TRF2 on telomeres by enhanced expression of GFP-TRF2 (Supplementary Fig. S3D), the formation of γH2AX foci during mitotic arrest was suppressed (Fig. 4D)^10^. Thus, mitotic telomere deprotection characterised by the loss of TRF2 from telomeres is initiated by caspase-dependent CAD endonuclease activity.

### DNA-dependent protein kinase (DNA-PK) is required for both γH2AX formation and TRF2 loss from telomeres during mitotic arrest

H2AX is phosphorylated on Ser139 during DDR by members of the phosphatidylinositol-3-kinase-related protein kinase family (PIKKs). We found that the formation of γH2AX foci during a prolonged mitosis was strongly inhibited by NU7441, a potent and selective inhibitor of DNA-PK^20^,^21^ (Fig. 4E). DNA-PK inhibition also prevented the release of TRF2 from mitotic telomeres (Fig. 4F). These results are consistent with a mechanism in which cleavage of telomere DNA by CAD generates double-strand breaks that activate DNA-PK, which then phosphorylates H2AX and enhances the removal of TRF2. By contrast, inhibition of the related kinase Ataxia Telangiectasia Mutated (ATM) with KU55933 (ATMi)^22^ did not significantly inhibit γH2AX formation or prevent TRF2 loss during mitosis (Fig. 4E,F). Consistent with other results, the caspase inhibitor zVAD-fmk inhibited both TRF2 loss from telomeres (Fig. 4E) and γH2AX formation (Fig. 4F) in these experiments.

### Caspase-dependent telomere damage during delayed mitosis controls subsequent cell fate

**Figure 5:**
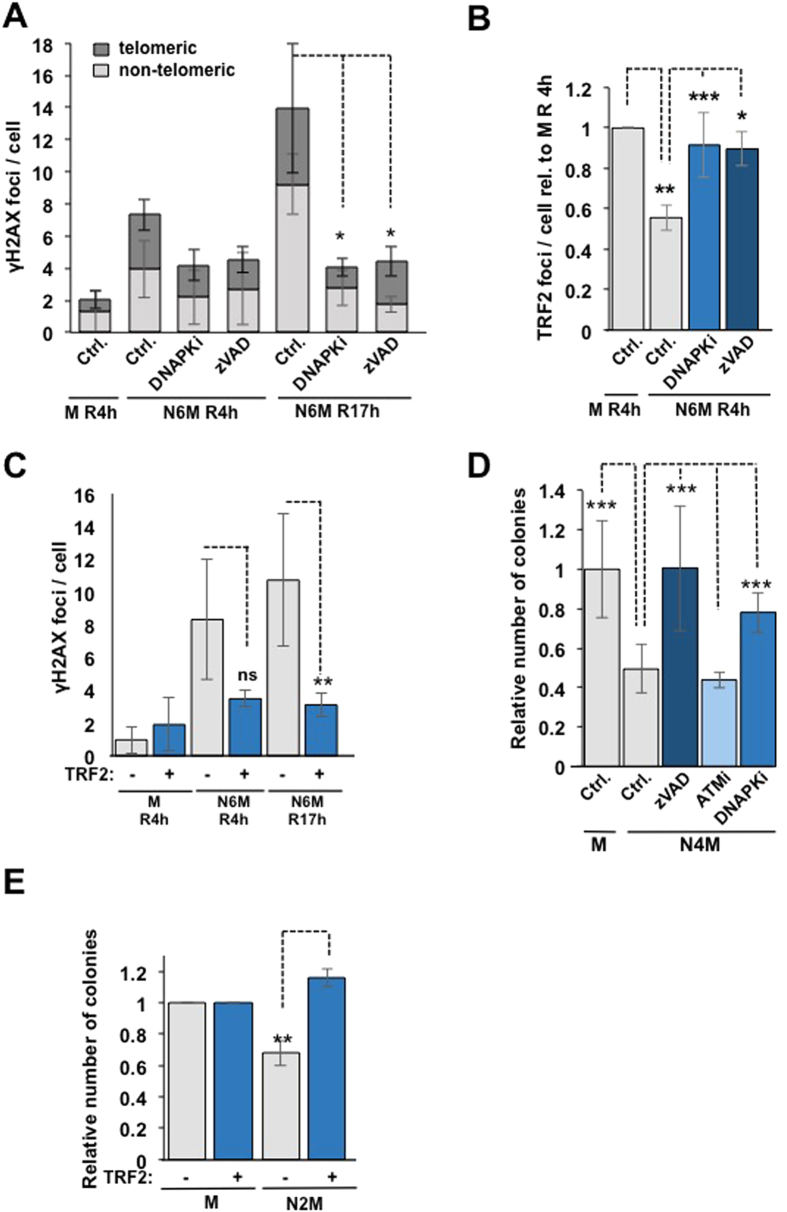
Effect of telomere damage during mitotic arrest on subsequent cell fate. (**A**) Mitotic telomere damage is amplified in interphase. U2OS cells were treated with nocodazole for 2 h then mitotic cells were replated in nocodazole containing medium +/− inhibitors for 4 h (N6M), washed and released in nocodazole-free medium for 4 h (R4h) or 17 h (R17h). Normal mitotic cells (M) were used as control. The graph shows data from three independent experiments. *p < 0.05. (**B)** Inhibition of DNA-PK or caspase-3/7 during mitotic arrest recovers the loss of TRF2 from telomeres that persists in interphase. U2OS cells were treated with nocodazole +/− DNA-PK inhibitor (NU7441, PKi) or zVAD-fmk (zVAD). The number of TRF2 foci per cell relative to the control is shown. *p < 0.05, **p < 0.01, ***p < 0.001. (**C)** Expression of GFP-TRF2 reduces the number of γH2AX foci in mitotic arrest. Cells in which expression of GFP-TRF2 was induced and un-induced control cells were treated with 250 ng/ml nocodazole and washed and re-plated as in (**A)**. The mean number of γH2AX foci per cell +/− SD from three independent experiments is shown. **p < 0.01. (**D**) Inhibition of caspase-3/7 or DNA-PK during mitotic arrest recovers the inhibition of subsequent cell proliferation and/or survival in clonogenic assays. U2OS cells were treated with 100 ng/ml nocodazole +/− chemical inhibitors of DNA-PK (NU7441, PKi), ATM (KU55933, ATMi) or Caspase-3/7 (zVAD-fmk, zVAD) for 2 h and then mitotic cells were washed off and re-plated in nocodazole containing medium +/− inhibitors for further two hours (N4M). The mean number of colonies surviving after 10–14 days +/− SD in three independent experiments is shown. ***p < 0.001. (**E**) Expression of TRF2 recovers the inhibition of cell proliferation and/or survival due to mitotic arrest in clonogenic assays. GFP-TRF2 cells +/− doxycycline (Dox) were treated with 100 ng/ml nocodazole for 2 h and then mitotic cells were washed off and re-plated. Normal mitotic cells are shown for comparison. The mean number of colonies surviving after 10–14 days +/− SD in three independent experiments is shown. **p < 0.01.

When cells were arrested in mitosis for a prolonged period and then released into interphase there was a strong increase in γH2AX foci at telomeric and especially non-telomeric sites (Fig. 5A), probably due to amplification of the initial telomere damage or DNA replication stress. Inhibition of DNA-PK or caspase-3/7 activity specifically during mitotic arrest prevented both the subsequent increase in γH2AX foci (Fig. 5A) and the loss of TRF2 from telomeres that otherwise persisted after mitotic arrest (Fig. 5B). Furthermore, GFP-TRF2 reduced the number of DNA damage foci in cells released from mitotic arrest (Fig. 5C). Thus, the initial caspase-dependent DDR, which is restricted to telomeres during mitosis, results in a more widespread DDR after release into interphase.

To determine the long-term effects of mitotic stress on cell proliferation and survival, we performed clonogenic survival assays. Inhibition of caspases or DNA-PK (but not ATM) during the period of mitotic arrest (Fig. 5D), or expression of GFP-TRF2 (Fig. 5E), rescued the inhibitory effect of mitotic arrest on subsequent cell colony formation. By contrast, inhibition of caspases immediately after release of cells from mitotic arrest failed to counter the inhibition of colony formation and inhibition of DNA-PK or ATM after mitosis strongly inhibited colony formation (Supplementary Fig. 4). Thus, inhibition of telomere damage responses specifically during mitotic arrest promotes subsequent cell survival, whereas DNA-PK and ATM play critical roles in interphase after mitotic stress.

### The role of caspase-dependent telomere damage during mitosis

**Figure 6:**
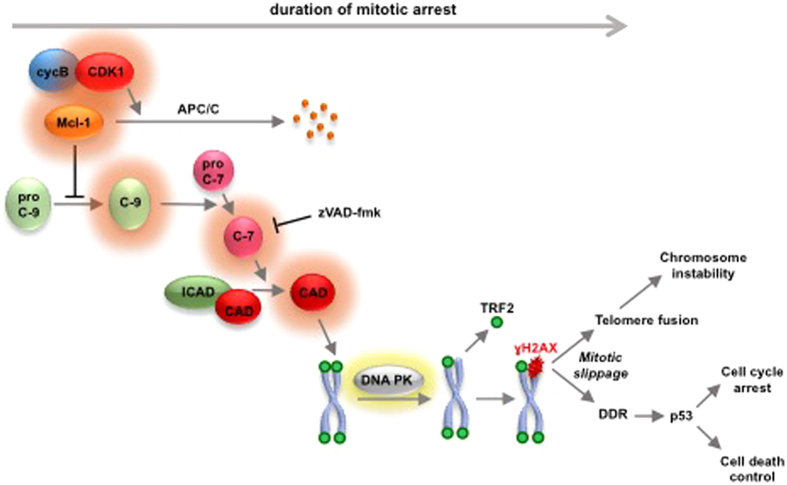
Mitotic arrest initiates a caspase-dependent telomere damage response. Prolonged mitotic arrest promotes the time-dependent destruction of Mcl-1 after its phosphorylation by CDK1-cyclin B. Mcl-1 loss promotes caspase activation and the activation of the endonuclease CAD, resulting in selective DNA double-strand breaks at telomeres, activation of DNA-PK, TRF2 loss from telomeres and formation of γH2AX. If caspase activation exceeds a threshold, it may result in apoptosis direct from mitosis, but if mitotic slippage caused by the slow destruction of cyclin B precedes this threshold, then cells respond in interphase through a full DNA damage response (DDR) and p53 activation. Activation of DNA repair mechanisms can cause telomere fusions leading to chromosome instability.

We have identified a mitotic stress pathway that causes telomeric DNA damage and controls subsequent cell fate and proliferation (Fig. 6). We propose that this pathway is initiated by the APC/C-dependent destruction of Mcl-1 during mitotic arrest^5^. Subsequent caspase-3/7 activation causes DNA damage at telomeres through the endonuclease CAD, which produces DNA strand breaks and selectively activates DNA-PK. This promotes TRF2 loss, resulting in telomere deprotection and the formation of telomeric γH2AX, most likely by direct phosphorylation of H2AX by DNA-PK.

The repair of DNA damage at telomeres by non-homologous end joining (NHEJ) is suppressed during mitosis^23^. However, if NHEJ is activated when cells exit mitosis with damaged telomeres, CAD-induced DNA double-strand breaks could result in telomere fusions and subsequent errors in chromosome segregation and duplication. These abnormalities could in turn cause further mitotic stress^24^. Caspase-dependent telomere damage might therefore initiate chromosome instability (CIN) in cells that survive a prolonged mitotic arrest. To counter these damaging effects, induction of a widespread DDR and p53 activation would normally limit further proliferation and induce cell death or cellular senescence. However, overexpression of Mcl-1 or other members of the Bcl-2 family that suppress the mitotic DDR, or the loss of p53 or other components of the downstream response^12^,^25^, could allow CIN cells to persist and acquire further loss or deletion of chromosomes, promoting a cancer phenotype.

## Material and Methods

### Reagents and antibodies

Reagents were purchased from Sigma-Aldrich unless specified. Drugs were obtained from Sigma-Aldrich (etoposide, actinomycin D, NU6027), Enzo Life Sciences (zVAD-fmk), Tocris (KU55933, NU7441, SB218078), or Selleck (Hesperadin). Primary antibodies were anti–γ-H2AX (pS139; Santa Cruz; sc-101696); anti-Actin (Sigma; A2066); anti-TRF2 (clone 4A794) (Millipore; 05-521); anti-caspase-3 (BD Transduction Laboratories; cat. 610322); anti-caspase-7 (Santa Cruz; sc-8510); anti-CAD (Thermo; PA5-19913). Secondary antibodies were HRP-linked anti-mouse, anti-goat or anti-rabbit (Biorad); Alexa-488–conjugated anti-mouse or anti-rabbit (Invitrogen); Alexa-594–conjugated anti-mouse or anti-rabbit (Invitrogen).

### Cells

U2OS (HTB96) cells were obtained from Cell Services, Cancer Research UK London Research Institute. A549 cells were obtained from ATCC (LGC Standards). GFP-TRF2, YFP-ICAD (D117E/D224E) and MAD2 under the control of a doxycycline–inducible promoter were stably integrated in U2OS Flp–In cells (Invitrogen).

### Cell synchronisations and treatments

U2OS cells were pre-synchronised in mitosis by washing-off rounded-up cells after a 2 h treatment 250 ng/ml nocodazole. Pre-synchronised cells were then either replated in nocodazole for a prolonged mitotic arrest or released in fresh media without nocodazole. Control mitotic cells were obtained by washing-off untreated asynchronous cultures and replating in fresh media.

Where stated, cells were treated with the following drugs (concentrations unless indicated otherwise in specific experiments): 50 μM etoposide, 500 nM actinomycin D, 20 μM z-VAD-fmk, 50 nM Obatoclax, 200 nM Navitoclax (ABT-263), 10 μM KU55933 (ATMi), 1 μM NU7441 (DNA-PKi, or PKi). For release experiments cells were treated as described above and the released into fresh, drug-free, medium in 8-well chamber slides (Ibidi). After the indicated times the now adherent cells were pre-permeabilised with 0.5% Triton X-100 in PBS for 3 minutes and processed as described below.

### Western blotting

For Western blots cells were lysed in 50 mM Tris/HCl (pH 7.4) containing 0.27 M sucrose, 1% (v/v) Triton X-100, 1 mM EDTA, and 0.1% (v/v) 2-mercaptoethanol with protease inhibitors (Roche). 10 μg of protein were loaded in each well. Membranes were blocked in 5% (w/v) non-fat milk in TBS 0.2% Tween-20. All primary antibodies were incubated overnight at 4 °C.

### Immunofluorescence assays and microscope image acquisition

γH2AX and telomere fluorescence *in situ* hybridisation (FISH) co-staining with a probe (5′-TTAGGG-3′) on metaphase spreads was performed as described previously^10^. Images were acquired using a Leica SP5II laser scanning confocal microscope with a HCX Pl Apo CS 63 × 1.4 lens. Co-localisation between γH2AX foci and telomeres was assessed in at least 25 cells per treatment. TRF2 foci and signal intensity were determined using ImageJ and a customized macro. Images were prepared using Adobe Photoshop and Illustrator software.

### Flow cytometry

At least 10000 cells per sample were analysed by flow cytometry (FACScan, BD Biosciences) and data were processed with FlowJo (Tree Star). Sub-apoptotic caspase 3/7 activity was analysed using DEVD-NucView 488 probe. To determine sub-apoptotic caspase 3/7 activity, mitotic cells were synchronised for 2 h with nocodazole and replated in the drug for indicated times with or without 5 μM DEVD-NucView 488 (Biotium) and indicated co-treatments. Samples were then collected at specified times and fixed in 2% paraformaldehyde in PBS for simultaneous analyses. Asynchronous cells were used as controls in parallel experiments. Sub-apoptotic and apoptotic populations were distinguished on Side Scatter/FL1 (NucView 488) dot plots. Finally, the mean fluorescence intensities in sub-apoptotic cells were related to background fluorescence in each sample and to a value measured in a sample from asynchronous cells set as 1.

### Clonogenic Survival Assay

U2OS cells or U2OS cells stably expressing GFP-TRF2 or GFP in a doxycycline-inducible manner were treated with 100 ng/ml nocodazole. Arrested cells were released into normal medium in 10 cm dishes at 5000 cells per dish. Between 10 and 14 days later, cells were washed with PBS, fixed, and stained with 2% Giemsa. The number of colonies on each plate was counted. Inducible cells were treated for 18 h with 1 μg/ml doxycycline prior to nocodazole treatment.

### siRNA transfections

For siRNA transfections U2OS cells were seeded at a density of 1 × 10^6^ cell/15 cm dish one day prior to transfection in 15 ml of medium. The next morning 5 ml of Optimem medium were mixed with 30 μl RNAiMAX (Invitrogen) transfection reagent and 10 nM siRNA duplex. siRNA Smart pools (Thermo Scientific) were used for initial experiments and individual oligos were obtained from MWG (Table 1). The transfection mix was incubated for 20 mins at room temperature before being added dropwise to the cells. The medium was changed in the evening and the cells were incubated in total for 36 hours before being split for experiments.

### Statistical analysis of data

Statistical analyses were conducted using Microsoft Excel. Differences between samples were assessed using Student’s t-test and p values were determined for 5% significance levels.

## Additional Information

**How to cite this article**: Hain, K. O. *et al.* Prolonged mitotic arrest induces a caspase-dependent DNA damage response at telomeres that determines cell survival. *Sci. Rep.* **6**, 26766; doi: 10.1038/srep26766 (2016).

## Supplementary Material

Supplementary Information

## Figures and Tables

**Table 1:** Oligonucleotides used for siRNA-mediated ablation of protein expression.

siRNA	Company	sequence
CASP7-01	MWG	GGGCAAAUGCAUCAUAAUA
CASP7-02	MWG	GAUCAGGGCUGUAUUGAAG
CASP7-03	MWG	UACCGUCCCUCUUCAGUAA
CASP7-04	MWG	CCAGACCGGUCCUCGUUUG
CASP7 pool	Thermo	pool of all 4 above
CASP3-01	MWG	CCGACAAGCUUGAAUUUAU
CASP3-02	MWG	CCACAGCACCUGGUUAUUA
CASP3-03	MWG	GAAUUGAUGCGUGAUGUUU
CASP3-04	MWG	GCGAAUCAAUGGACUCUGG
CASP3 pool	Thermo	pool of all 4 above
CAD-1	MWG	AAACGCACCAUCAUUCCUA
CAD-2	MWG	GAACCUGGAUCACAUAAUA
CAD-3	MWG	GCACGGAGCUGACGGAAGA
CAD-4	MWG	AGACAAGGUUGAAGCGGAA
TRF2 -1	MWG	GAACAAGCGGACAAUA
TRF2 -2	MWG	GCAAGGCAGCUACGGAAUC
TRF2 -3	MWG	GACAGUACAACCAAUAUAA
TRF2 -4	MWG	CCGAACAGCUGUGAUGAUU
TRF2 pool	Thermo	Pool of all 4 above
MCL1	MWG	GGACUUUUAGAUUUAGUGA

siRNA Smart pools (Thermo Scientific) were used for initial experiments and individual oligonucleotides were obtained from MWG. Pools of four individual siRNAs were used except for Mcl-1 as follows.
