# Supplementary material for: Prolonged mitotic arrest induces a caspase-dependent DNA damage response at telomeres that determines cell survival
Source: Sci Rep. 2016 May 27;6:26766. doi: 10.1038/srep26766 (PMC4882748; doi:10.1038/srep26766)

SUPPLEMENTARY INFORMATION

Prolonged mitotic arrest induces a caspase-dependent DNA damage response at telomeres that determines cell survival

Karolina O. Hain, Didier J. Colin, Shubhra Rastogi, Lindsey A. Allan and Paul R. Clarke*

SUPPLEMENTARY FIGURE LEGENDS

Supplementary Figure 1. Mitotic telomere damage is conserved and dependent on caspase-3/7 and DNA-PK.

A. Mitotic DNA damage is also present in a cell line expressing telomerase. A549 cells were treated with nocodazole for two hours and mitotic cells were washed off and collected (N2M) or replated in nocodazole for further 4 h (N6M). The cells were treated with nocodazole alone or also with zVAD-fmk or DNA-PKi (NU7441). Mitotic cells were spun onto glass slides and stained with an antibody against γH2AX and foci were counted.

B. Caspase-dependent mitotic DNA damage is due to the period of mitotic arrest and is independent of the use of a microtubule poison. U2OS cells with expression of MAD2 induced by doxycycline for 10-12 h were washed to remove mitotic cells, which were discarded, then 2 hours later additional mitotic cells were collected (2M) or replated for a further 4 hours (6M) +/- zVAD-FMK before samples were prepared for microscopy. For

comparison, cells without doxycycline-induction of MAD2 were treated with nocodazole for 2 hours and replated in nocodazole for further 4 hours (N6M).

Supplementary Figure 2. Sub-apoptotic caspase activation during mitotic arrest.

A. Schematic illustration of the detection of caspase-3/7 activity by NucView. The non-fluorescent DEVD-NucView 488 substrate is cleaved by active caspase 3/7, resulting in the nuclear migration and DNA binding of the NucView 488 dye, activating its fluorescent properties. This probe does not covalently bind and inhibit caspases allowing a sensitive and cumulative measurement of caspase activity.

B. Analysis of caspase-3/7 activity by flow cytometry. Asynchronous U2OS cells untreated (As) or pre-treated with actinomycin D for 24 h (ActD) were incubated with the fluorescent caspase-3/7 substrate DEVD-NucView 488 (NucView) or without substrate (no probe) for 4 h and analysed by flow cytometry. As depicted, sub-apoptotic and apoptotic cells were gated on the DEVD-NucView 488 / Side Scatter (FL1/SSC) channels for precision. Subapoptotic- and apoptotic-associated caspase 3/7 activity is indicated.

C. Cell cycle phase-dependent sensitivity to Navitoclax. Asynchronous cells (As) or cells arrested in mitosis for 2 h with nocodazole (N2M) were incubated with Navitoclax (Navi) at the concentration shown or zVAD-fmk (zVAD) for a further 4 h with further addition of nocodazole in the case of N2M samples. Samples were analysed by flow cytometry after incubation for 4h with DEVD-NucView 488 (NucView) and background fluorescence was acquired in samples not treated with the probe (Ctrl). The graph (right) shows relative mean fluorescence intensities in sub-apoptotic cells ± SD from 3 independent experiments, one of which is shown (left).

D. Time-dependent induction of sub-apoptotic caspase-3/7 activity by nocodazole is enhanced by Navitoclax. Asynchronous cells (As) or cells arrested in mitosis for 2 h with

nocodazole (N2M) were incubated with Navitoclax (Navi) alone or together with zVAD-fmk where indicated for a further 2, 4 or 8 h with further addition of nocodazole in the case of N2M samples. Upper panels: caspase-3/7 activity was assayed by flow cytometry using DEVD-NucView 488 (NucView). Control cells were incubated without NucView to obtain background fluorescence for each condition (Ctrl). Lower panel: relative mean fluorescence intensities ± SD from duplicate wells of a representative experiment.

Supplementary Figure 3. Role of TRF2 and CAD in telomere damage during mitotic arrest.

A. Caspase-dependent TRF2 loss is determined by the period of mitotic arrest. U2OS cells were treated with nocodazole for 2 h +/- zVAD-fmk. Mitotic cells were then washed off and either collected (N2M) or replated in nocodazole containing medium for a further 4 h (N6M). Mitotic cells were stained with antibodies against TRF2, γH2AX and a telomere probe (TTAGGG) and the number of foci per cell was counted. Scale bar, 10 μm.

B. TRF2 loss is not due to telomere loss. U2OS cells were treated with nocodazole for 2 hours and mitotic cells were washed off and collected (N2M) or replated in nocodazole for further 4 h (N6M). Some cells were also treated with zVAD-fmk at the same time as nocodazole (N6M zVAD). Normal mitotic cells (M) were also washed off from asynchronous cultures. Mitotic cells were spun onto glass slides and stained with a telomere probe and telomeric foci were counted. The graph shows the results from three independent experiments. Data were normalised to N6M samples.

C. siRNA depletion of CAD and TRF2. U2OS cells were transfected with siRNAs targeting CAD, TRF2 or CAD and TRF2 together. After 36 hours the cells were collected and lysed and processed for Western blotting with the indicated antibodies.

D. Overexpression of TRF2 prevents the formation of γH2AX foci during mitotic arrest. Mitotic U2OS cells expressing GFP-TRF2 or GFP alone treated with nocodazole were collected at indicated timepoints. Cells were spun onto microscopy slides and analysed for γH2AX foci. The graph shows mean numbers of foci per cell +/- SD from three independent experiments; * $p<0.05$.

Supplementary Figure 4. DNA-PK and ATM are required for cell survival after mitotic arrest. U2OS cells were treated with 100 ng/ml nocodazole for 2 h and then mitotic cells were washed off and re-plated in nocodazole containing medium +/- chemical inhibitors of DNA-PK (NU7441, PKi), ATM (KU55933, ATMi) or caspase-3/7 (zVAD-fmk, zVAD) for further two hours (N4M). The mean number of colonies surviving after 10-14 days +/-SD in three independent experiments is shown.

Supplementary Figure 1

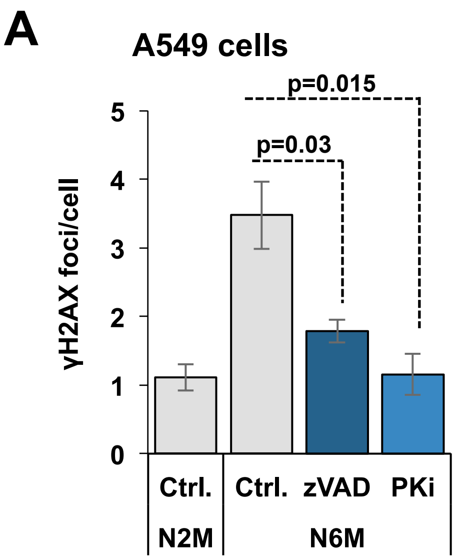

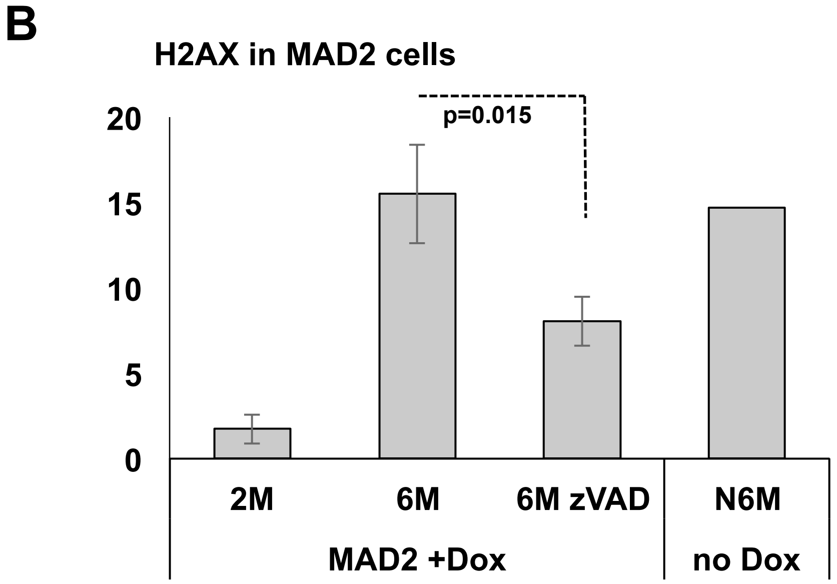

Supplementary Figure 2

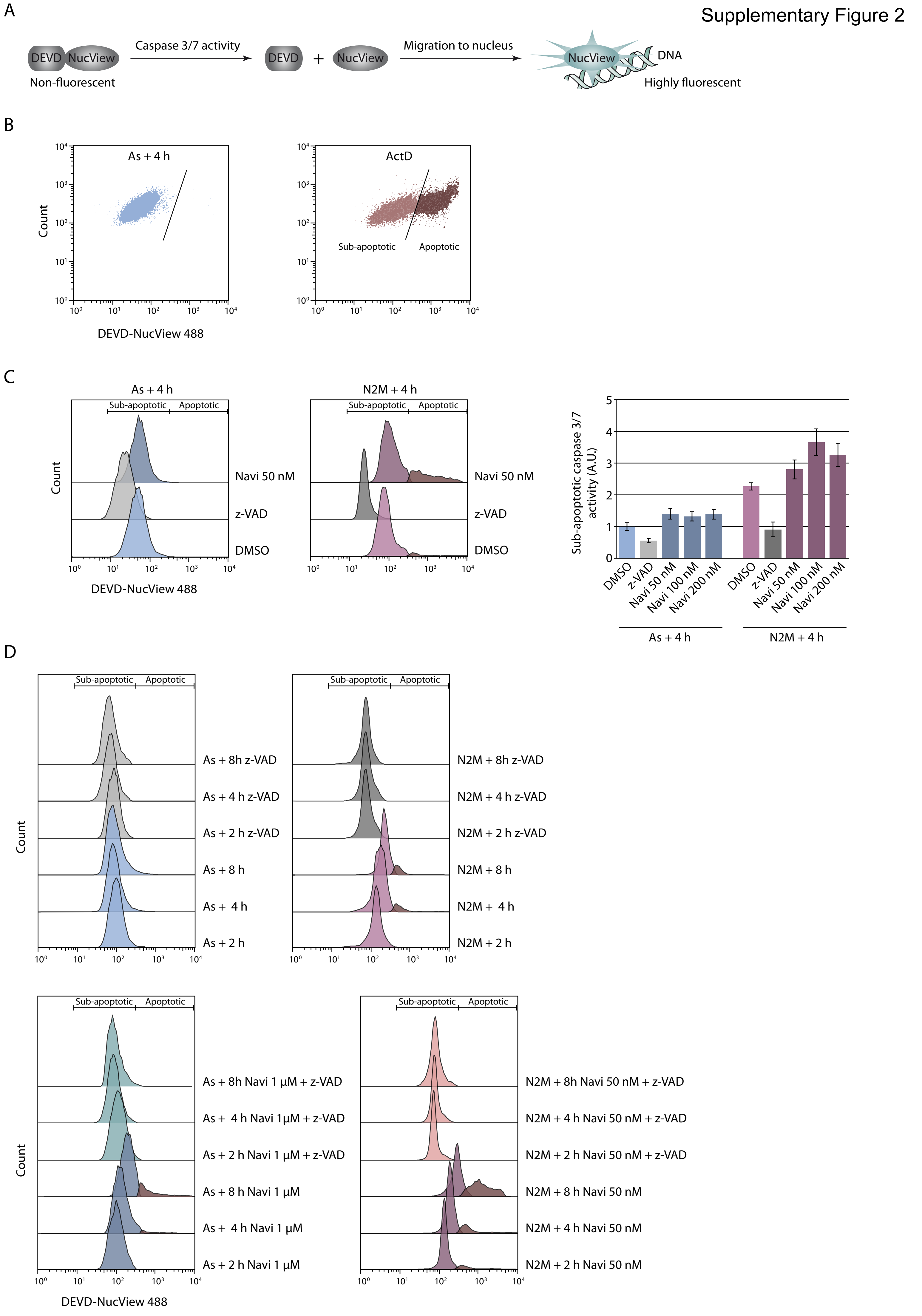

Supplementary Figure 3

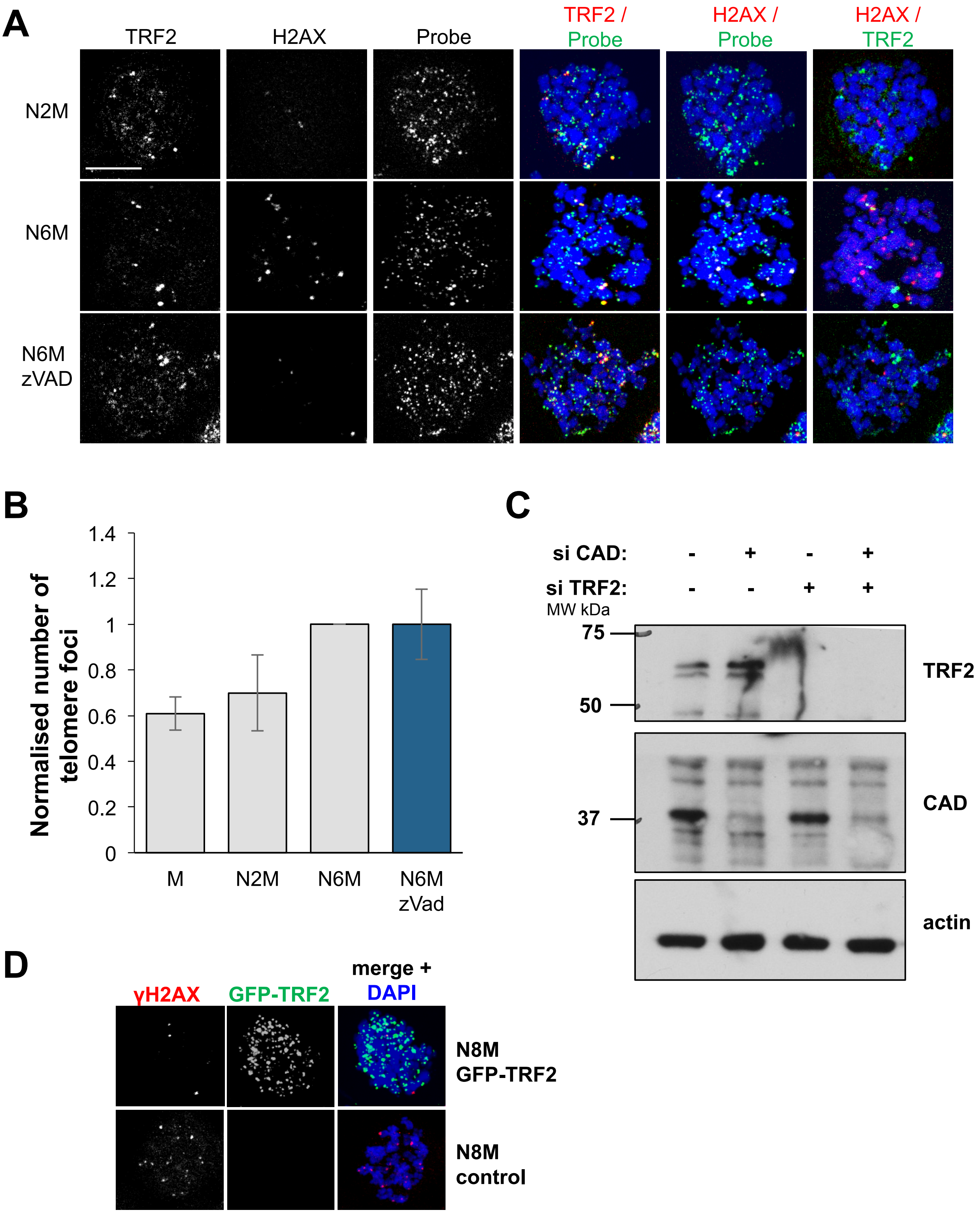

Supplementary Figure 4

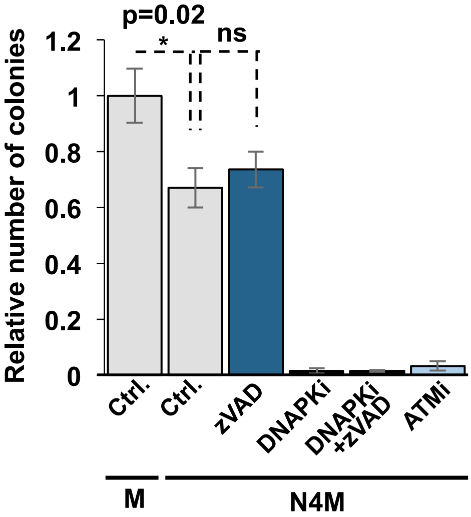

Supplement: Supplementary Information [file srep26766-s1.pdf]
